# Corrosion and Mechanical Behavior of Metal Materials

**DOI:** 10.3390/ma16030973

**Published:** 2023-01-20

**Authors:** Ming Liu

**Affiliations:** Center for Advancing Materials Performance from the Nanoscale (CAMP-Nano), State Key Laboratory for Mechanical Behavior of Materials, Xi’an Jiaotong University, Xi’an 710049, China; liuming0313@xjtu.edu.cn

Many high-strength metal-related materials and structures work under the coupling condition of harsh corrosion environments and complex loading [1,2,3,4,5,6,7,8,9,10,11,12,13,14,15,16,17], and related failure cases have been reported extensively all over the world. Hence, it is absolutely essential to investigate the corrosion and mechanical behavior of metal materials, aspects which mainly include corrosion fatigue [3,4,5], stress corrosion cracking [6,7,8], erosion corrosion [9], hydrogen-induced cracking [10], wear corrosion [18], etc. From the point view of materials and structures, failure can be caused by the unique mechanical and corrosive environment during their service life. The research methods of most forward environmental fractures [4,19,20] and the new mechanical analysis techniques for structures could all be useful in the study of those particular failure behaviors. Hence, this Special Issue, entitled “Corrosion and Mechanical Behavior of Metal Materials”, will mainly concentrate on how high-strength metal materials and structures work under the conditions of corrosion and complex loading.

The aim of this Special Issue is to discover the current state of the new methods, novel ideas, and advanced techniques of the related issues that link to the corrosion and mechanical behavior of metal materials. A wide range of research findings on different topics has been helpful in contributing to this Special Issue. The emphasis of these topics covers fundamental science and scientific problems that exist in engineering, experimental studies, analysis tools, numerical approaches, and design receipts. This Special Issue has the ambition to inspire and to disseminate the latest knowledge on the corrosion and mechanical behavior of metal materials and structures, laying the foundation for new ideas covering a range of topics for young researchers as well as leading experts in materials science and engineering and civil engineering.

The published papers covered in the topic area of this Special Issue encompass the corrosion fatigue characteristics of high-strength bridge steel, i.e., the degradation characteristics of galvanized and Galfan high-strength steel wire under marine corrosion and fatigue loading [21] and evaluating the corrosion fatigue degradation of the elastic center buckle of the short suspender of a suspension bridge under traffic loading [22]. The probabilistic seismic performance analysis of a corroded, reinforced concrete column and a corroded elastic bridge bearing was carried out by using the analytical model of the material degradation phenomenon. The seismic vulnerability of an aging bridge system was obtained by considering the failure functions of several related components [23]. Crucial attention was paid to the effect of severe plastic deformation on the corrosion behavior of a tantalum–tungsten alloy [24]; the severely deformed crystallographic orientations in the tantalum–tungsten alloy could be greatly weakened by an electrochemical process and could reduce the corrosion rate. The pre-exposure SCC (PESCC) of a ZK60 alloy induced by preliminary immersion in a NaCl-containing solution was systematically studied in one paper [25], and it was argued that the hydrogen stored within the corrosion product layer and the corrosion solution was responsible for the formation of these two zones. Meanwhile, the corrosion resistance of dilute Fe–Al alloys could be improved by preheating a nanoscale Al_2_O_3_ protective layer in a H_2_ atmosphere [26]. Similarly, a study on the corrosion behavior of a high-strength CuNi alloy in a harsh environment is also included within the scope of this Special Issue [27].
